# Beverage Consumption Habits in Italian Population: Association with Total Water Intake and Energy Intake

**DOI:** 10.3390/nu8110674

**Published:** 2016-10-26

**Authors:** Lorenza Mistura, Laura D’Addezio, Aida Turrini

**Affiliations:** CREA—Consiglio per la ricerca in agricoltura e l’analisi dell’economia agraria—Centro Alimenti e Nutrizione, Via Ardeatina 546, Rome 00178, Italy; laura.daddezio@crea.gov.it (L.D.); aida.turrini@crea.gov.it (A.T.)

**Keywords:** total water intake, energy intake, beverages

## Abstract

Background: The aim of this study was to investigate total water intake (TWI) from water, beverages and foods among Italian adults and the elderly. Methods: Data of 2607 adults and the elderly, aged 18–75 years from the last national food consumption survey, INRAN-SCAI 2005-06, were used to evaluate the TWI. The INRAN-SCAI 2005-06 survey was conducted on a representative sample of 3323 individuals aged 0.1 to 97.7 years. A 3-day semi-structured diary was used for participants to record the consumption of all foods, beverages and nutritional supplements. Results: On average, TWI was 1.8 L for men and 1.7 L for women. More than 75% of women and 90% of men did not comply with the European Food Safety Authority (EFSA) Adequate Intake. The contribution of beverages to the total energy intake (EI) was 6% for the total sample. Water was the most consumed beverage, followed by alcoholic beverages for men and hot beverages for women. Conclusion: According to the present results, adults and elderly Italians do not reach the adequate intake for water as suggested by the EFSA and by the national reference level of nutrient and energy intake. Data on water consumption should also be analyzed in single socio-demographic groups in order to identify sub-groups of the population that need more attention and to plan more targeted interventions.

## 1. Introduction

Total water intake (TWI) is essential for human health and life since it balances losses and assures adequate hydration of body tissues [1]. It is calculated that, of the total water intake consumed in a typical western diet, 20%–30% comes from food, and 70%–80% comes from beverages, but this may vary greatly among individuals depending on the diet they choose [2]. Current knowledge on water intake and its importance for the prevention of nutrition-based diseases is presented by Popkin [3]. Although good hydration is associated with reduction in urinary tract infections, hypertension, fatal coronary heart disease, various thromboembolis and cerebral infarct, all these results are not confirmed by clinical trials [3].

The role played by beverages in providing water in the diet has been recognised by international organizations such as the International Life Science Institute [4] and the European Food Safety Authority (EFSA) [2].

A recent document published by the World Federation of Hydrotherapy and Climatotherapy [5], attested and brought to the global attention the importance of water for certain body functions and the crucial role of appropriate hydration for overall health. Moreover, it represented a way to promote the inclusion of appropriate hydration as one of the goals of national and international health policies.

Drinking water during the day is of special importance for children and the elderly, the population groups that are vulnerable to dehydration. The elderly have been shown to have a higher risk of developing dehydration than younger adults. Modifications in water metabolism with aging and fluid imbalance in the frail elderly are the main factors to consider in the prevention of dehydration [6].

The EFSA Scientific Opinion on Dietary Reference Values for Water establishes that the dietary reference intake values for water should include water from drinking water (tap or bottled), all kinds of beverages, and from food moisture. The definition of adequate intakes (AI) proposed by the EFSA should be based both on observed intakes and on considerations of achievable or desirable urine osmolarity. Adequate total water intakes for females would have to be 2.0 L/day (P95 3.1 L) and for males 2.5 L/day (P95 4.0 L). The EFSA defines the same adequate intakes for the elderly as for adults [2].

The Italian Society of Human Nutrition has recently published the new reference values of adequate intake of water for the Italian population that do not vary from those defined by the EFSA [7].

Data on plain water and beverages intake are generally collected in national surveys. In order to study the patterns of beverage consumption of a population, it is not sufficient to report the average daily consumption of each beverage category. It is also essential to identify which variety of beverages are consumed and their contribution to total energy intake. In addition, the contribution of each beverage type to TWI permits an evaluation of the adequacy of drinking habits.

Methods adopted in dietary surveys differ from each other, and no standard method for the evaluation of water intake has so far been adopted [8]. This makes it difficult to compare results across and within countries.

In Italy, there are no recent studies focused on the TWI of the adult and elderly population. Recent published research regarded the consumption of energy drinks and alcohol among adolescents [9], of alcohol among adult and elderly men [10], and of caloric beverages among children and adolescents [11].

The present study aimed to investigate the TWI from plain water, beverages and foods and their contribution to overall water and energy intakes, among Italian adults and the elderly, by age group and gender. This analysis used dietary data from the INRAN-SCAI 2005-06 Study [12]. In addition, actual patterns of total daily water intake were compared with the AI recommended by the European Food Safety Authority.

## 2. Material and Methods

### 2.1. Study Population and Data Collection

The INRAN-SCAI 2005-06 survey was conducted on a representative sample of 1300 households randomly selected and stratified into the four main geographical areas of Italy (North-West, North-East, Centre, South and Islands) between October 2005 and December 2006. In total, 3323 (1501 males and 1822 females) individuals participated in the food survey, aged 0.1 to 97.7 years. A 3-day semi-structured diary was used. It is a mix of a specific format and free text where participants are able to record the consumption of all foods and beverages by meals, and nutritional supplements.

The food survey was conducted by a team of thirty well trained field workers. They met each subject three times during the survey, and carefully checked the food diaries and made specific questions to reduce errors such as misreporting and omissions (e.g., they asked if the participants took medicine and to remember to record the glass of water drunk for this purpose). In addition, to help the participants in recording the food and beverages consumed, they were given a picture booklet of the different standard portions for food and beverages.

In order to capture all the seasonal differences in intake, the sampled households were proportionally distributed among seasons (excluding Christmas and Easter periods): 25% in autumn, 25% in winter, 26% in spring and 24% in summer. In addition, the survey calendar was scheduled in order to take an adequate proportion of weekdays and weekend days at group level (78% and 22%).

Detailed information about the INRAN SCAI 2005-06 survey design, procedures, and methodologies can be found in the previous published papers [12,13].

For the present study, adults in the age range 18–75 years (*n* = 2607) were considered.

Data on nutrients intake, including water, were obtained using the updated version of the national food composition database [13]. In the case of foods and beverages that were fortified or enriched with one or more essential nutrients (including functional foods and special purpose foods), the nutrient content was retrieved at brand level from nutritional labels.

### 2.2. Plain Water and Beverages Consumption

Beverages were classified into eight categories: Hot beverages, including barley, coffee, tea and infusions; Milk and milk-based beverages; Fruit and vegetable juices; Caloric soft drinks, including soda and energy drinks and other sport drinks; Diet soft drinks, including beverages with sweeteners and without sugars; Alcoholic beverages (wine, beer and spirits); Water (tap and bottled); Other non-alcoholic beverages (soy based beverages and milk rice). Water added to recipes is included in the calculation of water coming from food.

### 2.3. Statistical Analyses

The 3-day mean of the total water intake (TWI) from the food and beverages categories previously described was evaluated for each subject. Mean values and standard errors of the food and beverages intakes were calculated for the 18–75 years old population and the sub-groups defined by gender and age classes (18–64 years and 65–75 years). Energy intake from beverage and food sources was also calculated.

The variety of beverage score was calculated as the average of the eight beverage categories on the three survey days.

To investigate the daily trend of the beverages consumption, the eating occasions were aggregated into main meals (breakfast, lunch and dinner) and snacks (morning, afternoon, after dinner).

The EFSA recommendations of water intake for each age and gender group were used to calculate the total shortfall in water consumption, and the proportion of adults who met or failed to meet the AI of water per day.

The Student’s *t*-test was applied to check whether there were differences in mean consumption of the TWI, water from food, water from beverages and total beverages consumption across subgroups of subjects defined by age and gender. The Mann–Whitney Test was performed for the eight beverages categories because their intakes were non-normally distributed. A two-sided p value of 0.05 was set to denote statistical significance.

All analyses were performed using the Statistical Analysis System computer software package (SAS package version 9.01; SAS Institute Inc., Cary, NC, USA).

## 3. Results

The total number of adults and elderly enrolled in this survey was 2607. Women represent 54% of the sample and the elderly, in the age class 65–75 years, represent 11% for both genders. The overweight/obesity rate is 49.7% and 38.4% for males and females, respectively.

**Figure 1:**
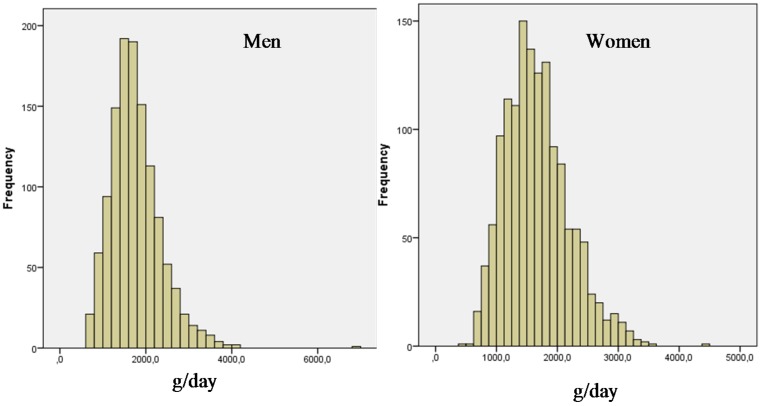
Frequency distribution of total water intake (g/day) over 3 days, by gender.

Total water intake (TWI) from all sources averages 1768.7 g/day for males and 1667.3 g/day for females (Figure 1).

**Figure 2:**
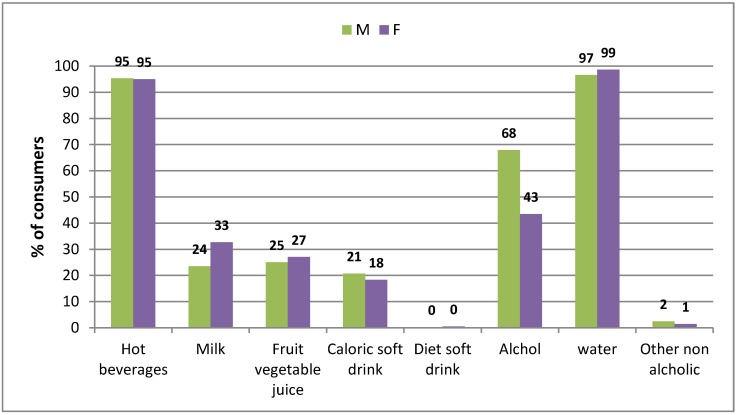
Popularity of beverages (% consuming over 3 days).

Beverages account for 56% of total water and 45% of the total weight of food and drink consumed (data not shown). Mean beverage consumption is 956 g/day (1015 g/day among men, 953 g/day among women). Water as a beverage is consumed by 97% of men and 99% of women and hot beverages are consumed by 95% in both genders, Figure 2.

Among men, alcoholic drinks are the second beverage category, contributing 8.9% to the TWI with 68% of consumers, followed by milk, fruit and vegetable juices and caloric soft drinks with similar percentages of consumers (25%, 24% and 21% respectively). Diet soft drinks and other non-alcoholic beverages are consumed by very few subjects of both genders. Alcoholic beverages are the third most consumed beverage category by women (43%), with a contribution of 3.4% on the TWI. The main sources of TWI are water for both sexes followed by hot beverages in females and alcoholic beverages in males (Table 1).

Mean total energy intake (EI) is 2137 kcal/day (SE 12.2). The contribution to the total EI from beverages is 6%. Alcoholic beverages are also the category with the greatest contribution to total energy intake, 4.5% in males and 1.9% in females. Caloric soft drinks contribute only 0.4% to total energy intake for the total sample (Table 1).

**Figure 3:**
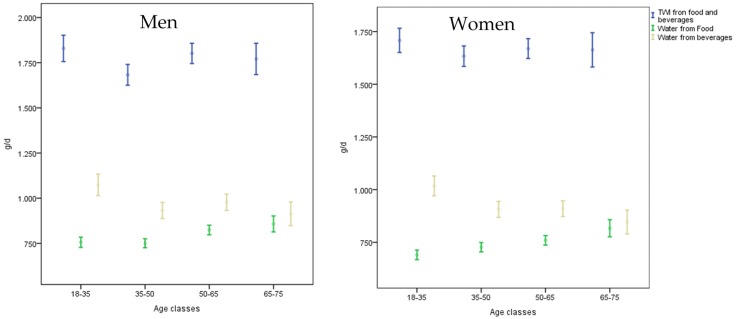
Mean and Confidence Interval (CI) of the Total Water Intake (TWI) from food and beverages, water from food and water from beverages by age classes and gender.

Table 2 presents the TWI, water intake from food and beverages, and the consumption of beverage categories. In Figure 3 the same variables are analysed by age classes in more detail. In general, water from beverages decreases with the increase of the age and the age class; 18–35 presents a significantly higher water intake from beverages than all the other classes in both genders (*p* < 0.001). Water from food significantly increases with age in both genders, and also in this case the younger age class has significantly lower intake than the others in both genders.

Among males, the mean intake of caloric soft drink and fruit and vegetable juice are significantly different by age class, even if there is a tendency to decline also in the other categories, except for alcoholic and hot beverages. Among females, the elderly drink significantly less and consume less water and caloric soft drinks than the younger participants.

The TWI correlated very highly with the weight of beverages and water from beverages (*r* = 0.90) and correlated more weakly with food weight (*r* = 0.65). Alcoholic drinks (*r* = 0.28), fruit and vegetable juice (*r* = 0.17) and caloric soft drinks (*r* = 0.15) have the highest correlation with energy intake. The mean variety score is 1.08, suggesting that adults and the elderly do not consume more than one type of beverage during the 3 days of the survey, out of the eight different beverage categories. There is a positive correlation with the TWI (*r* = 0.21) and energy intake (*r* = 0.30) (data not shown in table).

Beverage consumption has two peaks, for both gender and age classes, at lunch and dinner time. The younger males drink significantly more than older males in the evening during a nighttime snack, and the older females drink significantly less during lunch and dinner.

Finally, subjects who fulfill the EFSA AI recommendation of 2.5 L and 2.0 L are classified as criterion 1. Those who have the ratio of water/energy intake >1.0 are included as criterion 2 (it is considered as a value of 1 g of water per 1 kcal of energy intake). Those who meet both definitions (1 and 2) are classified as criterion 3. Following this analysis, the results show that more than 74% of women, and 90% of men do not comply the AI recommendation of consumption of water, as shown in Table 3.

## 4. Discussion

This study presented an analysis of total water intake from all food and beverages conducted on a representative sample of the Italian population aged 18–75 years from the INRAN-SCAI survey. Men had a higher TWI than women of both food and beverages, however, neither group met the EFSA recommended adequate intake for adult men (2.5 L) and women (2 L).

Although AI is used as a goal for individual water intake, water needs may vary a lot due to inter-individual variation [14]. When evaluating water intakes at individual level, several additional factors need to be considered which can influence water needs: physical activity; environmental factors, such as temperature; type of work; and other dietary factors, such as sodium intake.

Of all dietary sources of water, food was the leading one. Water content of food categories varies from 90% in fruit and vegetables to less than 5% of savoury snacks and confectionary [13].

Increasing the consumption of foods rich in water may have a positive effect both on hydration status and on dietary quality, although the effect of dietary water on hydration status is very poorly investigated [15].

The second main source of dietary water of Italian adults was plain water. Drinking plain water, tap or bottled, instead of any other beverage, permits the fulfillment of hydration requirements without providing energy, but providing small amounts of calcium, sodium and magnesium. Although the TWI can be increased in many ways, the most effective would be to increase the consumption of plain water [16]. Alcoholic beverages were the second and third beverage category most consumed by males and females respectively, with a high contribution to energy intake in men. Since the water content of alcoholic beverages can sensibly vary according to the type of beverage (for example, spirits vs. beer), the effect of their consumption on water balance varies accordingly, and increasing the strength and amount of drinks can result in a loss of fluid intake, rather than an increase [15].

Similar studies on TWI were conducted in France [17], in the United States [18], in Britain [19], and more recently in Spain [8] where the values for the TWI for both genders were very close to our results—in the study conducted in Spain, 68% of the TWI came from beverages, as opposed to 56% in Italy, and consequently the contribution of all beverages to energy intake was higher (12.2 vs. 5.6%). Like in Italy, in Spain the water intake from food increased with age and the water from beverages decreased; this trend was consistent with the recent study on vulnerability to dehydration of older people [20]. In the United States the contribution of beverages to energy intake was quite higher than in Italy, going to 22% among the 20–50 years old age group, and 14% among the ≥71 years old age group [18].

The consumption of caloric soft drink was higher for younger age classes (40.8 g/day 18–35 age class) and decreased with age (6.8 g/day 65–75 age class) which, compared to the Spanish study, is very low (96.2 g/day for all samples). Consequently, the contribution to energy intake is also very low (0.4%) when compared to the Spanish [8] results (6.1%), although this data also included children and adolescents. The US results were 5.7% for 20–50 year olds, 3.5% for 51–70 year olds and 2.1% for the elderly.

According to these results, beverages including plain water were consumed only during meals, and this may suggest an underreporting, especially of plain water drunk outside the meals. This is probably due to the survey tool in which the participants had to report the water consumption during meals, and snacks between meals, and the glass of water drunk out of the standard meals may have not always been recorded. The presence of a specific question about the water consumption could help the participants to record all water drinking occasions, even those not linked to the meals. Even regarding the consumption of alcoholic beverages, an underestimation in recording the consumption occasions is likely to occur because of the belief, due to cultural reasons, that drinking is regarded as socially undesirable [21,22].

The limitations of the food consumption survey, INRAN SCAI 2005-06, are addressed by Leclercq et al. [12]. Moreover, it is well known that the self-reported dietary record produces a general underreporting of consumption [23]. This was not taken into account in the consumption estimates.

## 5. Conclusions

The present analysis is, to our knowledge, the first attempt to explore total water intake among Italian adults and the elderly, based on data coming from the national dietary survey. According to the present results, adults and elderly Italians do not reach the adequate intake for water suggested by the EFSA and by the national reference level of nutrient and energy intake. Therefore, data on water consumption should also be analysed in single socio-demographic groups in order to identify sub-groups of population that need more attention and to plan more targeted interventions

## Figures and Tables

**Table 1:** Contribution of food and beverages to total water and energy intake.

		Contribution to Water Intake (g/Day)			Contribution to Energy Intake (kcal/Day)		
		Male	Female	Total	Male	Female	Total
All food and drink	Mean (SE) *	1768	1668	1714	2381	1931	2138
		16.9	14.0	11.0	18.5	14.0	12.2
Food only	%	44%	44%	45%	93%	95%	94%
		0.3%	0.3%	0.2%	0.1%	0.1%	0.10%
Beverages only	%	56%	56%	56%	7%	5%	6%
		0.3%	0.3%	0.2%	0.1%	0.1%	0.1%
Hot beverages	%	8%	8%	8%	0%	0%	0%
		0.2%	0.2%	0.1%	0%	0%	0%
Milk	%	2%	3%	2%	1%	1%	1%
		0.1%	0.1%	0.1%	0.1%	0.1%	0%
Fruit and vegetable juice	%	1%	1%	1%	1%	1%	1%
		0.1%	0.1%	0.1%	0.0%	0.0%	0.0%
Caloric soft drink	%	2%	1%	1%	1%	0%	0%
		0.1%	0.1%	0.1%	0.0%	0.0%	0.0%
Diet soft drink	%	0.0%	0.0%	0.0%	-	-	-
		0.0%	0.0%	0.0%			
Alcoholic Beverages	%	9%	3%	6%	5%	2%	3%
		0.3%	0.2%	0.2%	0.1%	0.1%	0.1%
Water	%	33%	38%	35%	-	-	-
		0.4%	0.4%	0.3%			
Other non alcoholic beverages	%	0%	0%	0%	0%	0%	0%
		0.0%	0.0%	0.0%	0.0%	0.0%	0.0%

* SE = standard error.

**Table 2:** Total water intake, water from food and beverages and beverage consumption (g/day), by gender and age group.

		Male			Female		
		18–64 (*n* = 1068)	65–75 (*n* = 134)	*p*	18–64 (*n* = 1245)	65–75 (*n* = 160)	*p*
TWI from food and beverages	Mean (SE)	1768 (18.2)	1771 (44.0)	n.s. *	1669 (15.0)	1663 (41.1)	n.s. *
	CI	1732.7–1804.0	1683.8–1857.8		1639.1–1697.9	1582.0–1744.5	
Water from food	Mean (SE)	778 (7.9)	856 (22.2)	0.001 *	727 (6.6)	817 (20.6)	0.000 *
	CI	762.6–793.5	813.6–901.5		713.8–739.9	776.3–857.6	
Water from beverages	Mean (SE)	990 (14.5)	913 (33.2)	n.s. *	942 (12)	846 (28.6)	0.007 *
	CI	961.7–1018.8	847.5–979.0		918.1–965.2	789.8–902.8	
Total beverages consumption	Mean (SE)	1024 (14.9)	945 (33.8)	n.s. *	965 (12.1)	867 (29.1)	0.006 *
	CI	995.1–1053.5	878.4–1012.0		940.7–988.4	810.2–925.0	
Hot beverages	Mean (SE)	135 (3.3)	139 (13.8)	n.s. ^§^	138 (3.2)	129 (9)	n.s. ^§^
Milk	Mean (SE)	38 (2.8)	39 (6.7)	n.s. ^§^	49 (2.4)	53 (7.4)	n.s. ^§^
Fruit vegetable juice	Mean (SE)	27 (2.3)	16 (4.8)	0.003 ^§^	28 (1.9)	23 (4.3)	n.s. ^§^
Caloric soft drink	Mean (SE)	33.(2.7)	7 (2.5)	0.000 ^§^	22 (1.7)	9 (2.8)	0.000 ^§^
Diet soft drink	Mean (SE)	1 (0.8)	0 (0.0)	n.s ^§^	1 (0.3)	0 (0.0)	n.s. ^§^
Alcoholic Beverages	Mean (SE)	163 (6.0)	184 (15.1)	n.s. ^§^	58 (2.7)	66 (8.3)	n.s. ^§^
Water	Mean (SE)	624 (12.9)	559 (31.9)	n.s. ^§^	667 (11.1)	588 (26.4)	0.022 ^§^
Other non alcoholic	Mean (SE)	1 (0.9)	1 (0.5)	n.s. ^§^	2 (0.5)	0 (0.3)	n.s. ^§^

* *t*-test comparison with a significant level 0.05; ^§^ U of the Mann–Whitney test comparison with a significant level 0.05; CI, Confidence Interval.

**Table 3:** Combined classification for the total water intake (TWI) following established criteria.

Criteria Classification	Men (*n* = 1202)	Women (*n* = 1405)
Criterion 1: % (*n*)	10.6 (127)	23.9 (336)
Criterion 2: % (*n*)	13.3 (160)	27.0 (379)
Criterion 3 (1 and 2): % (*n*)	5.5 (66)	14.9 (210)

EFSA: European Food Safety Authority. (1) Criterion 1: TWI >2.5 L men, >2 L women (aged 14 to 75 years); (2) Criterion 2: Ratio of total water intake and total energy >1; (3) Criterion 3: Both criteria 1 and 2.
